# Stigma mastery in people living with HIV: gender similarities and theory

**DOI:** 10.1007/s10389-021-01480-7

**Published:** 2021-03-15

**Authors:** Charles Patrick Namisi, John C. Munene, Rhoda K. Wanyenze, Anne R. Katahoire, Rosalinda M. Parkes-Ratanshi, Stella Kentutsi, Maria M. Nannyonga, Robina N. Ssentongo, Mabel K. Ogola, Maria Sarah Nabaggala, Geofrey Amanya, Agnes N. Kiragga, Richard Batamwita, Nazarius M. Tumwesigye

**Affiliations:** 1 The Ugandan Academy of Health Innovation and Impact, Infectious Diseases Institute, College of Health Sciences, Makerere University, P.O.Box 22418 Kampala, Cassia Hill Road, 4th Floor, Mckinnell Knowledge Centre, Kampala, Uganda; 2 School of Public Health, College of Health Sciences, Makerere University, P.O.Box 7072, Kampala, Upper Mulago Hill Road, Mulago, Kampala +256, Uganda; 3 PhD Programme, Makerere University Business School, Plot 21A, Port Bell Road, Kampala, Uganda; 4 Institute of Public Health Cambridge University, Cambridge, UK; 5 The National Forum of People Living with HIV Networks in Uganda or NAFOPHANU, P.O.Box 70233, Plot 213, Sentema Road, Mengo, Kampala +256, Uganda; 6 Nsambya Home Care Department, St Francis Hospital, Nsambya, P.O.BOX 7146, Kampala, Nsambya Hill Road, Kampala +256, Uganda; 7 Kitovu Mobile Limited, P.O.Box 207, Masaka, Plot 4 & 10 Delhi Road, Masaka +256, Uganda; 8 Catholic Agency for Overseas Development or CAFOD, PO Box 66153, Nairobi, Plot 209/75/75, Vihiga Road, Nairobi, +254, Kenya; 9 Infectious Diseases Institute, College of Health Sciences, Makerere University, P.O.Box 22418, Cassia Hill Road, 4th Floor, Mckinnell Knowledge Centre, Kampala +256, Uganda; 10 FHI 360 Uganda, P.O Box 5768, Kampala, Plot 15 Kitante Close, Kampala +256, Uganda

**Keywords:** HIV-related, stigma, gender, mastery, similarities, theory-building

## Abstract

**Aims:**

This study aimed to determine the prevalence of, factors associated with, and to build a theoretical framework for understanding Internalsed HIV-related Stigma Mastery (IHSM).

**Methods:**

A cross-sectional study nested within a 2014 Stigma Reduction Cohort in Uganda was used. The PLHIV Stigma Index version 2008, was used to collect data from a random sample of 666 people living with HIV (PLHIV) stratified by gender and age. SPSS24 with Amos27 softwares were used to build a sequential-mediation model.

**Results:**

The majority of participants were women (65%), aged ≥ 40 years (57%). Overall, IHSM was 45.5% among PLHIV, that increased with age. Specifically, higher IHSM correlated with men and older women “masculine identities” self-disclosure of HIV-diagnosis to family, sharing experiences with peers. However, lower IHSM correlated with feminine gender, the experience of social exclusion stress, fear of future rejection, and fear of social intimacy. Thus, IHSM social exclusion with its negative effects and age-related cognition are integrated into a multidimensional IHSM theoretical framework with a good model-to-data fit.

**Conclusion:**

Internalised HIV-related Stigma Mastery is common among men and older women. Specificially, “masculine identities” self-disclose their own HIV-positive diagnosis to their family, share experiences with peers to create good relationships for actualising or empowerment in stigma mastery. However, social exclusion exacerbates series of negative effects that finally undermine stigma mastery by young feminine identities. Thus, stigma mastery is best explained by an integrated empowerment framework, that has implications for future practice, policy, and stigma-related research that we discuss.

## Introduction

People living with HIV (PLHIV) differ in stigma experienced, and some have none at all. In some PLHIV, society rejects them as gay, or sex workers, and others just because they are women or young, hence intersecting stigma (Goffman 1963; Meyer 2003; Turan et al. 2019). Stigma surveys show that nearly half of PLHIV internalise the public’s negative stigma as their own. The majority of PLHIV are young adults (18–49 years), and they commonly (34–90%) avoid close relations (Arnold et al. 2016; NAFOPHANU 2019; Simbayi et al. 2015). Concealing HIV identity triggers distress, as self-disclosure is part of intimate social interactions, where a successful social identity is a married person with children (Earnshaw et al. 2015; Sjåstad et al. 2020). Further, stigma deters individuals from seeking HIV testing, treatment, and prevention behaviours along the HIV care cascade, making it difficult to achieve the 90-90-90 targets (WHO 2018). Hence, powerlessness is a central part of intersecting stigma in young PLHIV. However, social deviants can transform from an earlier identity to an HIV self-concept to enjoy protection from stigma.

Nearly half of PLHIV do not have stigma (NAFOPHANU 2019; Simbayi et al. 2015; Stigma Survey UK 2015). These men and women of the same age share skills, attitudes, and behaviours or age-related changes. These changes create social identities for control of internal stigma, and some of them even transform others to lead normal lives (Pearlin et al. 1981), what we called *internalised HIV-related stigma mastery* (IHSM). However, strategies to eliminate stigma and discrimination at a global level seem ineffective in low-resource countries (UAC 2015; UNAIDS 2014). Hence, the current global trend is towards interventions for intersecting stigma (Turan et al. 2019; Van Brakel et al. 2019). However, critical practice gaps remain: (1) the sequences of nearly half of PLHIV mastering their structural, relational, and emotional stigmas have not been elaborated, and (2) stigma models put minimum emphasis on integrating protective factors at multiple levels (Goffman 1963; Jackson-Best and Edwards 2018; Stangl et al. 2019). Finally, ten countries, including Uganda, accounted for the majority of new HIV infections in sub-Saharan Africa (UNAIDS 2020). Uganda had a generalised (6.2%) HIV positivity among adults (MoH 2017). Hence, defining the mastery of structural, interpersonal, and individual stigmas or ecological perspective in a theoretical model is central to zero stigma globally.

## Theoretical framework for mastering stigma

Social stigma shaping individuals’ experiences is a well-known concept. Goffman (1963), in his seminal work on management of spoiled identity, and later Link and Phelan (2001) defined the social structures that reinforce power inequalities for stigma (Goffman 1963; Link and Phelan 2001). People with past experience or fear of rejection conceal their HIV identities, which adds distress and strains relational intimacy that undermines their stigma mastery practices. However, nearly half of PLHIV have no internal stigma (NAFOPHANU 2019; NAP+ 2014; Simbayi et al. 2015; Stigma Survey UK 2015); these men and women use the support of peers and families to foster skills to resist stigma (Firmin et al. 2017; NAFOPHANU 2019). Therefore, the dynamic process of power inequalities perpetuates structural stigma. Thus, this cross-sectional survey was conducted to determine the prevalence of and factors associated with stigma mastery, which were used to define the theoretical model underlying stigma mastery for adults living with HIV. Notably, multiple statistical analyses were used to define and validate the theoretical model, whose implications for future stigma-related research and practice are discussed.

## Materials and methods

### Study design

A cross-sectional quantitative study was conducted to determine the prevalence and factors associated with IHSM, on the basis of which a theory was generated.

### Study setting and population

The study was conducted at St Francis Hospital, Nsambya Home care department, a community-based HIV care clinic that had 6940 PLHIV. This site, together with Kitovu mobile, initiated an “Evidence-based responsive HIV Stigma Reduction Project” in Uganda in 2014. The project aimed to reduce internalised stigma by 30% in 12 months among a cohort of 2018 participants in central and southern Uganda. A sample of 666 participants was nested in a cohort of PLHIV at St Francis Hospital, Nsambya, in central Uganda. Inclusion criteria were age 18 years or older, PLHIV, and informed consent to participate in the study.

### Sample size and procedure

Sample size calculation was based on assumptions of 80% power, 95% confidence interval, and prevalence of internalised HIV-related stigma of 50% (NAFOPHANU 2013) to detect a 10% difference between men (50%) and women (60%) using multiple regression. Stratified random sampling with proportional allocation to women and age groups representative of the study PLHIV and resampling methods were used to randomly select the 666 participants.

### Data collection tool and technique

The *PLHIV Stigma Index* version 2008 (Stigma Index) was used for measurements. The Stigma Index documents and provides evidence for advocacy in implementation of stigma reduction interventions. The index had been used in over 100 countries, including Uganda, by December 2017 (IPPF 2008). The overall internal consistency of the index was acceptable (α = 0.72); the subscales scored social stigma (0.73), self-stigma (0.62), and disclosure concerns (0.49) (LNP+ 2012).

As part of full community partnership, the team selected data clerks (80% PLHIV) with experience in using smartphones who were trained for 5 days on data entry using an electronic form of the PLHIV Stigma Index version 2008. Data clerks pretested the questionnaire for data collection on the fifth day of training. Their feedback resulted in minor changes to the final questionnaire. The data collection process lasted 14 days in November 2014. The process involved face-to-face interviews in English or “Luganda”, a local dialect, lasting 45 to 60 minutes per participant. Participants’ responses were entered as codes into the electronic form and double-checked for completeness by a supervisor before submitting the data to a central server.

### Measurement of variables

The *dependent variable, IHSM*, was measured as “none” of the feelings of internal stigma on the PLHIV Stigma Index. The anchor statement was “In the last 12 months, have you experienced the following feelings: *blame self, blame others, feels sinful, guilt, suicidal, low self-esteem, feels ashamed, or none*? Respondents chose “yes” = 1 or “no” = 0. According to the calculation, the cumulative score was 0–8.

The *independent variables* were as follows: (1) socio-demographic variables including male sex, age group under 40 years, marital status, education, household location, and employment, coded “Yes” = 0 or “No” = 1; (2) experience of discrimination [anchor statement: in the last 12 months, how often have you been “excluded from the following activities” or “fearful of the following”, etc.], coded 1 = never to 4 = often. We recoded “never” = 0 and “ever” = 1; (3) work-related; (4) rights, policy, and power to influence; (5) giving support to other PLHIV; (6) HIV testing, diagnosis, and treatment; (7) disclosure and confidentiality: “How would you describe the reactions of the following people when they first knew you were living with HIV?” 1 = very discriminatory to 6 = not applicable. We recoded 1 = “supportive” and 0 = “none supportive”; stress was reverse-coded. Finally, variables were transformed into a new composite variable of social exclusion, social stress, fear of future rejection and social intimacy, and HIV disclosure family support.

### Statistical analysis

Statistical analysis, in increasing complexity, was used to select the best variables for the IHSM model. IBM SPSS Statistics version 24 software was used in preliminary analysis and factor analysis to inform the use of AMOS 27 software with structural equation modelling to confirm (Arbuckle 2013) in three phases.

*Preliminary data analysis* was conducted in three steps. *First*, data were screened for assumptions of logistic regression. Missing data were less than 5%. Redundant items were dropped to achieve tolerance of = .647 to .834 (closer to 1), variance inflation factor 1.198 to 1.545 (below 3.3), and minimal multicollinearity (Hair et al. 2014). *Second*, bivariate correlation between variables was used to select variables with *p* ≤ 0.20, which were entered into a generalised linear model. Backward stepwise regression was used to reduce 38 variables to an eight-variable model that predicted IHSM. *Third*, crude adjusted prevalence ratios and their 95% confidence intervals were calculated to avoid overestimation of relative risk between two variables. This simple parsimonious model guided decisions during the factor analysis phase.

*Examination of the validity and reliability phase* to specify the factor structure (dimensionality), relationships, and theoretical model underlying the IHSM model was carried in seven steps: *First*, our large sample (*N*= 666), with subject-to-variable ratio (27:1), factor loadings (≥.6), and Kaiser-Meyer-Olkin (≥.50) and Bartlett’s test of sphericity .124 to .574 (*p* ≥.001), all suggested sample adequacy for factor analysis (Hair et al. 2014). *Second*, a zero-order correlation matrix of covariates for IHSM showed 23 out of 36 (63.9%) significant relationships. Two clusters of highly correlated variables were observed. *Third*, to specify the underlying IHSM factor structure highly correlated variables, exploratory factor analysis with principal component analysis was used to extract factors from variables. *Fourth*, varimax rotation with principal axial factoring with oblimin rotations were used. *Fifth*, eigenvalues greater than 1.0 and percentage of variance explained were used to select factors for retention. *Sixth*, two to five items were loaded (> 0.60) on each factor. The highest-loading items were used to identify and interpret the factors for the specified 25-item/8-factor theoretical IHSM model.

*Seventh*, confirmatory factor analysis was used to validate the 25-item/8-factor theoretical model-to-data fit. Standardised path coefficients fixed at 1.0, *p* values and their absolute, incremental, and parsimonious goodness-of-fit indices were estimated for the measurement model (Hu and Bentler 1999). A bootstrapping method was used to test the indirect effects of intervening variables between independent and outcome variables. Indirect effects were claimed if zero was not included in the lower and upper bounds of calculated 95% bias-corrected bootstrap confidence intervals from 5000 bootstrap replicates. Finally, multiple fit indices were used to achieve levels of acceptance for measurement model validity. Internal reliability measures were Cronbach’s alpha of each factor = .60–.84 (≥ 0.60), but our model is multidimensional, so the average variance extracted for each construct was equal to .513 to .734 (≥ 0.50), and all construct reliability values (.73–.93) exceeding (>.70) were used (Hair et al. 2014). Our results indicated that all variables had good convergent validity on factors. Square roots of the average variance extracted for two constructs were greater than the correlation between their respective constructs, evidence that supported good discriminant validity (Fornell and Larcker 1981). Thus, our 25-item/8-factor multidimensional measurement model was reliable and valid.

Finally, for *phase*, *face*, and *nomological validity* of the IHSM model, the relationships of constructs were linked to an existing empowerment framework and social reality of significant patterns that affirmed explanations in the model that we present.

## Results

The majority of participants were female (65%), aged 40 years or older (56.9%), and had known their HIV-positive status for over 5 years (68.5%). Age groups were distributed as follows: 2.0% were 18–19 years, 4.2% were 20–24 years, 7.1% were 25–29 years, 29.9% were 30–39 years, 35.7% were 40–49 years, and 21.2% were ≥ 50 years, which was representative of the study population. Further, experiences of negative psychological effects were common: stress (91 %), fear of verbal insult (69 %), fear of sexual intimacy (69 %), and awareness of being gossiped about (58%). Also, families were supportive (61%), and participants gave support to peers (52%). However, the actual experience of social exclusion was uncommon (4.5%). Thus, participants were largely women, in middle adulthood, who commonly experienced and perceived psychosocial effects during social interactions.

### Prevalence of internalised HIV-related stigma mastery

Our first objective was to determine the prevalence of IHSM. Overall, 303 out of 666, or 45.5% [95% CI (41.7–49.3), mean 1.20 (SE = .060 and SD = 1.553), skewness 1.535 (<2), kurtosis 2.050 (≤ 7)] reported IHSM. Further, other participants endorsed a number of internal feelings of stigma, whose percentages are shown in brackets: one (24.3%); two (13.5%); three (6.3%); four (5.6%); five (2.6%); six (1.2%); seven (0.9%); and all eight (0.2%). Thus, nearly half of the participants endorsed none of internal stigma feelings.

### Factors associated with internalised HIV-related stigma mastery

The second objective was to determine the factors associated with IHSM.

**Fig. 1:**
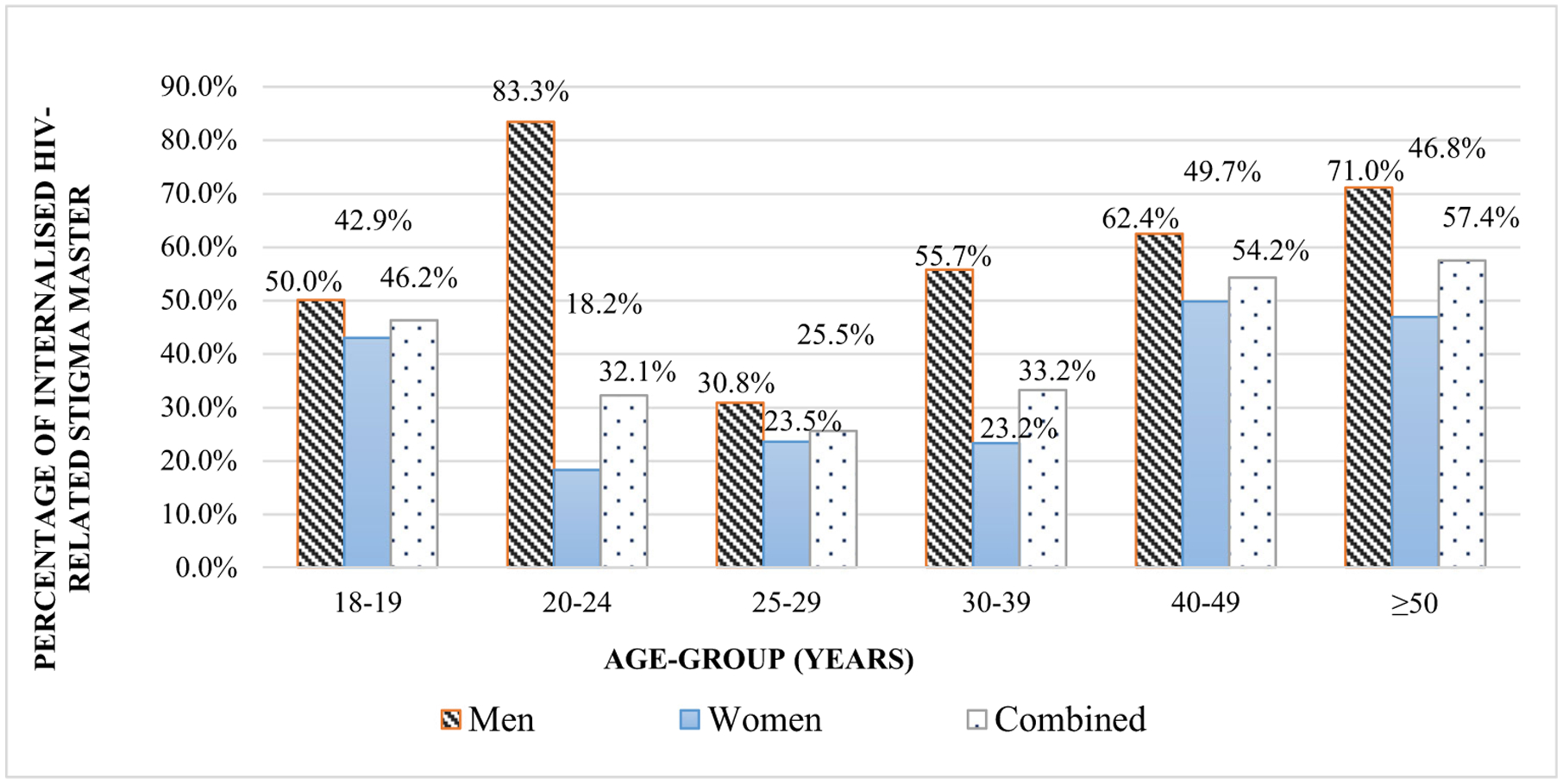
Age-group-specific prevalence of internalised HIV-related stigma mastery by gender for 666 people living with HIV, 2014. *Note*. Lines across represent men, plain women, and dots for combined gender

Age-group-specific prevalence of IHSM by gender is shown in Fig. 1.

IHSM increased with age for both women and men (Fig. 1), implying that men and women in early adulthood follow similar trajectories in IHSM. Then we compared the eight feelings of internal stigma by gender (Table 1).

Bivariate correlation between participants’ feelings of internal stigma and gender is shown in Table 1.

Men compared to women were twice as likely to endorse having none of the feelings of internal stigma (Table 1), while women were more likely than men to report feelings of self-blame, blame for others, shame, and guilt. Thus men seemingly had better stigma mastery. Therefore, socio-demographic and psychosocial factors for IHSM were stratified by gender and age group (Table 2).

Men and women reported similar negative psychological effects. Further, discriminatory experiences and feelings of power to influence programs were constant across age groups (Table 2). Our results imply gender similarities in experiences of negative psychological effects of stigma and age-related experiences of discrimination and feelings of power; hence, the need to control for socio-demographic and psychosocial variables for predicting IHSM.

### Multivariate analysis

A multivariate regression model for independent factors that predicted IHSM is given in Table 3.

Four variables, i.e. being male, higher stress, supportive family at HIV disclosure, and giving support to others, predicted higher odds of IHSM, while three variables. i.e. young age, fear of verbal insults, and fear sexual intimacy, predicted lower odds of IHSM (Table 3).

However, IHSM was common (>10%), so odds ratios overestimated relative risk. Therefore, the prevalence ratios were calculated for IHSM, adjusting for various covariates stratified by gender and age 40 years (Table 4).

After stratifying by gender and age 40 years, IHSM was more prevalent in older men (124%) with supportive family at HIV disclosure, and women (50%) who gave support to peers (Table 4), while IHSM was less prevalent (45–85%) across all participants with fear of verbal insults. Thus, age-related changes, gender, social support, and anticipated aggression predict stigma mastery. Therefore, exploratory factor analysis defined the structure underlying the IHSM model.

### Defining the theoretical model underlying internalised HIV-related stigma mastery

The third objective was to define the theoretical model underlying IHSM. Three steps were used: (1) correlation analysis to identify groups of variables with high correlations; (2) exploratory factor analysis to specify the factor structure (dimensionality) underlying variables with high correlations and theoretical model; (3) confirmatory factor analysis to validate the theoretical model fit to data.

### Correlation analysis between covariates and IHSM

Without controlling for any variables “zero-order” correlation matrix for the eight covariates for IHSM (Table 5).

All eight covariates correlated significantly with IHSM in three clusters: first, social exclusion with its negative effects on IHSM; second, giving peers support had higher correlations with gender and age compared with IHSM, a *suppressor effect* (Table 5). Finally, disclosure family support, age, and gender. Our results indicate that negative effects of social exclusion, interact with disclosure behaviours, and social identities to predict stigma mastery. Thus, exploratory factor analysis was used to specify the underlying factor structure of the IHSM model.

### Specifying the underlying factor structure of IHSM model

Factors, factor loadings, percentage of variance, and reliability statistics for exploratory factor analysis using principal component analysis with varimax rotation on IHSM items were used to specify the model structure (Table 6).

Social exclusion, gender, giving support to others, and HIV disclosure to family (antecedents) influenced social stress, fear of future rejection, fear of social intimacy, and age-related processes (mediating) that in turn predicted IHSM (consequence) (Table 6). Hence, IHSM is a 25-item, eight-construct multidimensional model.

### Validating the stigma mastery structural model

**Fig. 2:**
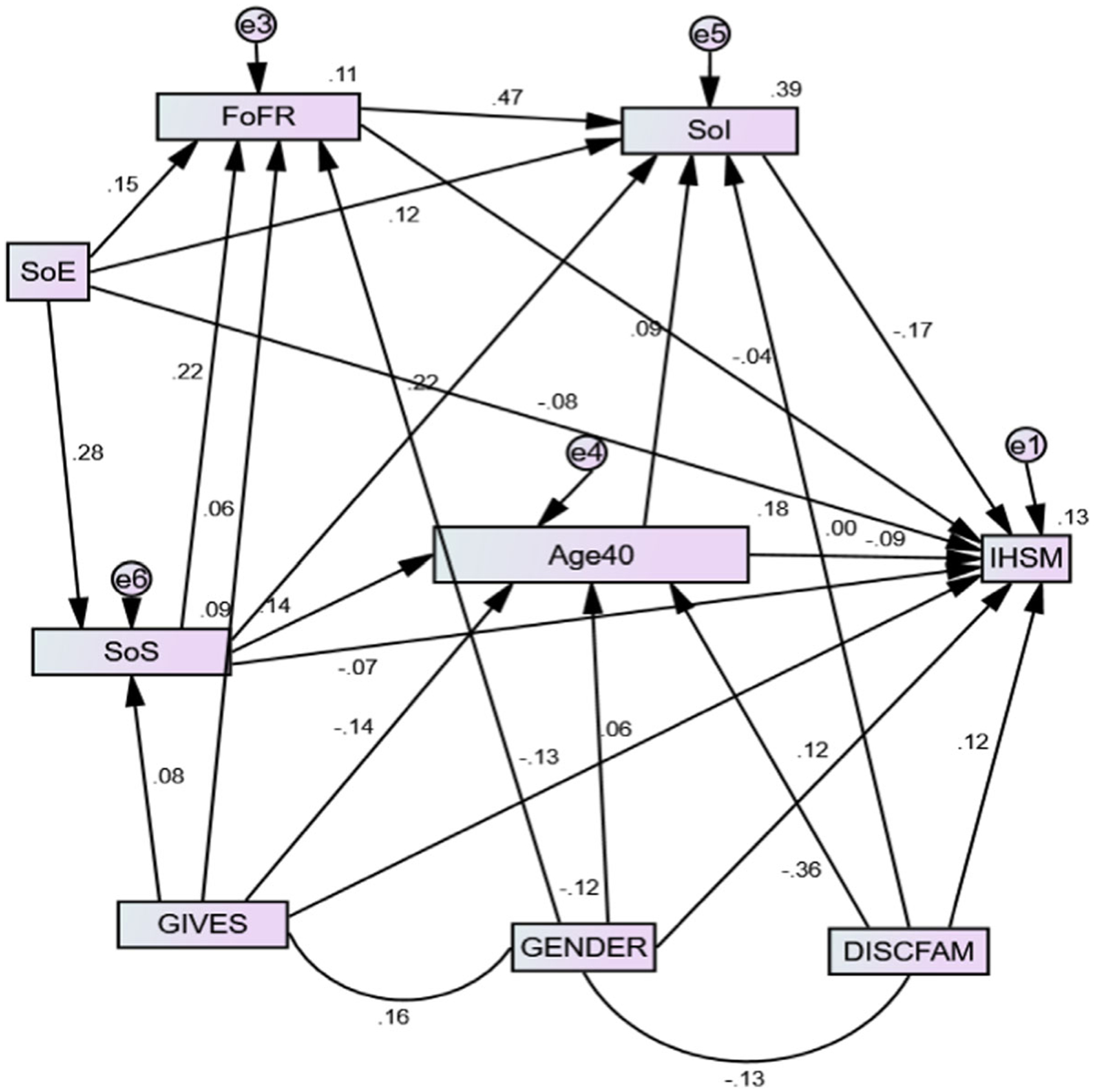
Structural equation model for predicting internalised HIV-related stigma mastery. χ^2^ = 16.955, *df* = 11, *p* = .109 (≤ .05); root mean square error of approximation (RMSEA) = .029, 90% CI [.000, .054] (≤ .06); GFI= .99, comparative fit index (CFI) = .99 (≥ .95); Tucker-Lewis Index = .97 (≥ .90); chi-square divided by degrees of freedom (CMIN/df) = 1.5 (1–5). *Note*. Age40 is age using a cut-off of ≤ 40 years. FoFR = fear of future rejection, GIVES = giving support to others, IHSM = internalized HIV-related stigma mastery, SoE = social exclusion, SoI = fear of social intimacy, SoS = social stress. Gender is in reference to men. χ^2^ =16.955, df = 11, p =.109 (≤ .05); Root Mean Square Error of Approximation (RMSEA) =.029, 90%CI [.000, .054] (≤ .06); GFI= .99, Comparative fit index (CFI) =.99 (≥ .95); Tucker-Lewis Index =.97 (≥ .90); Chi-square divided by degrees of freedom (CMIN/DF) =1.5 (1–5)

Confirmatory factor analysis/structural equation model with path analysis, unstandardized and standardized regression coefficients, and fit indices to judge the hypothesized theoretical relationships that predicted IHSM are shown in Fig. 2 and Tables 7 and 8.

The IHSM measurement model had a good model-to-data fit (Hu and Bentler 1999), with good convergent and discriminant validity with factors (Fig. 2 and Table 6). Overall, the model explained 13.3% of the variance in IHSM, a medium effect size. Further, the variance accounted for by each factor was as follows: fear of social intimacy (39.1%), age-related changes (17.8%), fear of future rejection (10.8%), and social stress (8.7%). Thus, the results validated IHSM as a multidimensional theoretical measurement model.

### Total, direct, and indirect effects of constructs in the stigma mastery model and their interpretation

Pathways by which individuals master stigma using total, direct, and indirect effects with standardized regression coefficients are shown in Fig. 2 and Table 7, with 95% bias-corrected bootstrap confidence intervals (Table 8).

A structural model supporting 21 direct and 16 mediated hypotheses is shown in Fig. 2 and Tables 7 and 8. All direct effects were significant except for: stress (*p* = .104), fear of future rejection (*p* = .392), and giving peers support (*p* = .088) each on IHSM (Table 7). Giving peers support had suppression effects on IHSM. Mediation analyses (Fig. 2 and Table 8) are summarised under two themes: social exclusion undermines IHSM and age-related changes enhance IHSM.

#### Social exclusion and negative psychological effects undermined stigma mastery (Model H1a)

The hypotheses that social exclusion would directly and indirectly predict IHSM were supported. Specifically, past experience of social exclusion by family directly undermined IHSM (β = −.08, *p* < .05) and triggered social stress (β = .28, *p* < .001), fear of future rejection (β = .15, *p* < .001), and fear of social intimacy (β = .12, *p* < .001). Social stress (β = .22, *p* < .001), fear of future rejection (β = .47, *p* < .001), and younger (18–40 years) age (β = .09, *p* < .01) each predicted fear of social intimacy. Further, men were older (< 40 years) (β = −.12, *p* < .05), and had less fear of future rejection (β = −.13, *p* < .001). Finally, fear of intimacy undermined IHSM (β = −.17, *p* < .001).

#### Mediation analysis of social exclusion, negative psychological variables, stigma mastery

When social stress was included as a mediator variable in the model (c = .041, *p* < .001), the direct effects of social exclusion on age-related changes dropped to zero. After fear of social intimacy was added as a mediator variable (c = .081, *p* < .001), the direct effects of fear of future rejection on IHSM were no longer significant (c′ = −.038, *p* = .437). Further, once age-related changes, fear of future rejection, and fear of social intimacy were included as sequential mediators in the model (c = −.079, *p* < .001), the direct effects of social stress on IHSM were no longer significant (c′ = −.066, *p* = .145), and when negative psychological variables were included as sequential mediators (c = −.163, *p* < .001), the direct effects of social exclusion on IHSM were no longer significant (c′= −.083, *p* = .05). Negative psychological variables partially explained how many predictors undermined stigma mastery. In sum, past experience of social exclusion via a sequence of social stress, age-related changes, fear of future rejection, and fear of social intimacy (the *negative psychological effects*) explains why feminine women had stigma. *Model 1a* suggests that social exclusion by family exacerbates sequences of negative effects that eventually undermine stigma mastery practices in feminine women.

#### Gender, disclosure behaviours, giving support, age-related changes in stigma mastery (Model H1a)

The hypotheses that age-related changes would directly and indirectly predict stigma mastery were supported. Specifically, early adulthood (18–40 years) directly predicted IHSM (β = −.09, *p* < .05), along with family support at HIV disclosure (β = −.36, *p* < .001), giving support to peers (β = −.14, *p* < .001), and male gender (β = −0.12, *p* < .001).

#### Mediation analysis for gender, giving support, psychological effects, and age-related changes in IHSM

When age-related changes were added as a mediator variable in the model (c = −.031, *p* < .01), the direct effects of HIV disclosure family support on fear of social intimacy dropped to near zero. After the fear of social intimacy and age-related changes were added as sequential mediator variables (c = .017, *p* < .05), the direct effects of giving support to peers on fear of future rejection dropped to zero. Again, age partially explained why HIV disclosure family support enhanced IHSM (c = .039, *p* < .05) and men actualized IHSM (c = .028, *p* < .05). Finally, after fear of future rejection was included as a mediator variable in the model (c = −.073, *p* < .001), the direct effects of gender on fear of social intimacy dropped to zero. *Model 1b* shows that social exclusion drove men and older women to use age-related changes for self-disclosure of HIV to families and to create supportive environments for giving support to peers as they achieved mastery of stigma (*masculine identity*). Thus, men and older women used positive masculine identity to actualise stigma mastery.

## Discussion

Our results confirm that stigma mastery is common among men and older women living with HIV. Two tenets, social exclusion with its negative effects and age-related changes, define stigma mastery. Specifically, social exclusion drives men and middle-aged women with “masculine identity” to disclose their HIV status to their supportive families and peers for actualising stigma mastery practices. Thus, integrating social exclusion and social cognition into an empowerment framework may have wider implications for practice and stigma-related research, as we discuss.

### Prevalence of and factors associated with internalised HIV-related stigma mastery

IHSM is common among PLHIV. Specifically, nearly half (45.5%) of our representative sample, which included two-thirds women and two-thirds middle-aged adults, reported IHSM. IHSM was twice as prevalent in men as in women, though men were significantly older than women. Further, both men and women gained IHSM during the ages of 18 to 40 years, or “early adulthood”. Our IHSM figure lies within a range of 5–65% across cultures of PLHIV (Arnold et al. 2016; Emlet et al. 2014; NAFOPHANU 2019; NAP+ 2014; Stigma Survey UK 2015). However, unlike earlier stigma surveys, we linked our findings to a theoretical model that had a good fit to the data (Fig. 2). Thus, significant correlates for stigma mastery were demographic and psychosocial factors that we discuss under two themes: social exclusion with its negative effects, and age-related changes in stigma mastery.

### Social exclusion exacerbates negative psychological effects to undermine stigma mastery

Our sample suggests that social exclusion has both direct and indirect negative effects on stigma mastery (Model 1a). Specifically, the fear of social shame associated with people living with HIV (PLHIV) leads families to reject them. Further, past experience or fear of social exclusion triggers sequences of distress and fear of further rejection by family that ultimately undermine stigma mastery. Moreover, women in our sample endorsed significantly higher self-blame, shame, and guilt than men. Uganda’s male-dominated society teaches women to express their emotions, and they blame younger PLHIV, usually women, for their immoral sexuality. These reasons combined partly explain why young women are powerless against social stigma as they remain trapped in fear of rejection by family.

It is well known that fear of rejection harms relationships (Downey et al. 1998; Pearlin et al. 1981; Sjåstad et al. 2020). Women reporting more stigma than men is also a known relation (Simbayi et al. 2015; Stigma Survey UK 2015), which we linked to their young age and powerlessness to recognize structural stigma (Loutfy et al. 2012; Stets and Burke 2014). On the other hand, older people have better control of their own emotions (Stets and Burke 2014) such as stigma mastery practices. Hence, our results illustrate pathways through which structural stigma, with its negative relational effects, ultimately undermines stigma mastery among feminine women. On the other hand, men and older women with age-related changes are more likely to have achieved stigma mastery.

### Gender- and age-related changes drive stigma mastery

Age-related changes directly and indirectly predicted IHSM (Model 1b). Specifically, both men and women, throughout early adulthood, gain stigma mastery. These age-related changes are personal resources for individuals to disclose their HIV status to their families, build good relationships, and share experiences with peers as they actualize stigma mastery. Ugandan patriarchal society teaches men to control their emotions and to provide care to others. Further, women with age and resources achieve a masculine identity. Moreover, our study site has expert clients who enhance peers in self-care. Thus, this partly explains the participants’ use of positive masculinity to enhance stigma mastery.

The reduction in stigma in middle adulthood is a well-known relation (Emlet et al. 2014; Loutfy et al. 2012), which we linked to gaining mastery. People gaining mastery over their affairs is a well-known empowerment theory (Pearlin et al. 1981; Rappaport 1987). Gaining emotional control is another well-studied effect (Bussey and Bandura 1999; Crum et al. 2013), specifically the gender similarity hypothesis (Hyde 2005; Hyde 2014). Finally, adults narrating stories to peers create good relations for positive emotions (Deci and Ryan 2008; Thomas and Velthouse 1990), meaning in life, and good mental health (Inagaki and Eisenberger 2016). Thus, men and women use age-related changes for mastering stigma.

### Theory-building grounded in the data

Our sample suggests that participants responded to social exclusion (antecedents) in two different ways: young feminine women experienced fear of future rejection by close relations (mediating) that undermined their stigma mastery (Model 1a). This model aligns with social exclusion theory, which posits that social rejection is a major cause of anxiety (Baumeister and Tice 1990; Sjåstad et al. 2020). On the other hand, men and middle-aged women used positive “masculine identity” to disclose their HIV status to their families and give support to peers (antecedents) as personal resources (mediating) for actualising stigma mastery (consequence) (Model 1b). Model 1b aligns with social cognitive theory, which posits that individuals and their social cognition and behaviours influence each other (Bandura 1999). Thus, integrating social exclusion and social cognitive perspectives creates an improved empowerment framework that best explains the stigma reduction practices in our sample (Bandura 1999; Baumeister and Tice 1990; Thomas and Velthouse 1990). Alternative model are resilience and stress-related growth; however, resilience lacks a theoretical explanation, while stress-related growth (Park and Helgeson 2006; Tedeschi and Calhoun 2004) has been criticised for its inconclusive relationship with negative effects on well-being (Rzeszutek 2018). Hence, our contribution of a multidimensional empowerment framework integrates mastery in a sequence of psychosocial factors at multiple levels, whose practical implications for stigma-related research are discussed.

### Practical, research, and policy implications

Our findings suggest that practitioners can use mastery as a stigma-reducing intervention for powerless individuals. First, they must self-disclose their HIV-positive status to their families as a prerequisite for mastering control. Then gender-sensitive, age-appropriate sharing of experiences with peers can allay distress to overcome power inequalities. Hence, our mastery can guide stigma reduction practice, research, and policy at clinic and during positive health, dignity and prevention community dialogues (UNAIDS, GNP+ 2013).

#### Methodological strengths

A representative sample of PLHIV and advanced analyses were used to select the best model-to data fit. Our statistical model specified theories to eliminate measurement errors in explaining the stigma reduction practices. The practice integrating risk and protective factors at multiple levels was linked to an empowerment framework which allows for generalization of findings to stigma reduction practice in similar populations.

#### Study limitations

Self-reporting as a measure of self-stigma is subjective and a common method bias; however, it was minimised with full community partnership throughout the project. We together designed the study and collected data using 80% with or affected by HIV, who understood stigma. We then conducted analysis, wrote the report, and disseminated findings to stakeholders.

#### Future research

Future researchers can validate the claims of this preliminary mastery theoretical model that explains sequences of power relations at different stigma levels. Further, since the stigma resistance subscale has the lowest internal consistency, gender and social cognition at the structural and individual levels for stigma are barely measured using empowerment at multiple levels. Thus, researchers need to consider our assertions and at least four feelings of internal stigma, i.e. self-blame, shame, guilt, and sinfulness, for the next measures such as the PLHIV Stigma Index 3.0.

## Conclusions

In conclusion, we have shown that internalized HIV-related stigma mastery is high among men and older women (“masculine” people) living with HIV. Specifically, power inequalities around HIV identity tend to cause self-stigma to remain in young feminine identities. However, masculine identities use age-related changes to self-disclose HIV to their families and share experiences with peers as they build good relationships for actualising stigma mastery practices. Hence, integrating social exclusion and social cognitive processes into an improved empowerment framework best explains stigma mastery practices. We hope that mastery as an empowerment approach gains wider application in stigma reduction.

## Figures and Tables

**Table 1:** Correlation between participants’ feelings of internal stigma and gender for 666 people living with HIV, 2014

Feelings of internal stigma	Male (*n*) %	Female (*n*) %	Total (*n*) %	Chi-square *df* = 1	*p* value
I feel ashamed	41 (17.6)	112 (25.9)	153 (23.0)	5.85	0.016
I feel guilty	28 (12.0)	81 (18.7)	109 (16.4)	4.95	0.027
I blame myself	51 (21.9)	151 (34.9)	202 (30.3)	12.09	0.001
I blame others	13 (5.6)	91 (21.0)	104 (15.6)	27.39	0.000
I have low self-esteem	32 (13.7)	65 (15.0)	97 (14.6)	0.20	0.656
I feel I should be punished	8 (3.4)	23 (5.3)	31 (4.7)	1.20	0.276
I feel suicidal	7 (3.0)	23 (5.3)	30 (4.5)	1.88	0.177
I feel sinful	21 (9.0)	54 (12.5)	75 (11.3)	1.81	0.180
None	143 (61.4)	160 (37.0)	303 (45.5)	36.47	0.000

None of the internal feelings of stigma = “internalised HIV-related stigma mastery”, our dependent variable; *df*= degrees of freedom

**Table 2:** Socio-demographic and psychosocial variables associated with internalised HIV-related stigma mastery by gender and age group, 2014

Variables	Gender					Age group							
	Men	Women	Total	χ^2^	*p* value	18–19	20–24	25–29	30–39	40–49	≥50	χ^2^	*p* value
Socio-demographic													
IHSM: Yes	61.4	37.0	45.5	36.47	.000*	46.2	32.1	25.5	33.2	54.2	57.4	37.17	.000*
Gender: female						53.8	78.6	72.3	69.3	64.3	56.0	10.79	.056
Age: ≥ 40 years	63.1	53.6	56.9	5.59	.018*				30.7	69.3		34.86	.000*
Marital status: Single/separated/widow(ed)	35.2	71.8	59.0	84.04	.000*	100.0	82.1	57.4	54.8	57.6	59.6	16.97	.005^†^
Maximum education level: above primary	23.6	39.0	33.6	16.15	.000*	15.4	21.4	31.9	40.7	31.5	31.9	8.99	.109
Self-employed: No	48.5	52.2	50.9	0.83	.363	100.0	89.3	59.6	57.8	44.1	37.6	48.62	.000^†^
Children < 14 years/household: ≥ 3	37.8	23.6	28.5	15.00	.000*	61.5	0.0	21.3	27.1	29.8	34.8	22.23	.000^†^
Household location: rural/small town	22.3	40.6	34.2	22.60	.000*	76.9	67.9	66.0	68.3	75.6	70.9	4.07	.540
HIV testing													
Duration with diagnosis: ≥ 5 years	63.9	71.1	68.6	3.63	.057	76.9	60.7	38.3	55.8	76.9	83.7	58.96	.000*
Reason for HIV test: referred suspected TB	73.4	83.6	80.0	9.89	.002*	61.5	78.6	87.2	83.9	81.1	72.3	11.61	.040*
Pre- & post-test HIV counselling: No	21.0	12.0	15.2	9.58	.002*	23.1	32.1	6.4	18.6	11.3	15.6	14.26	.014*
Psychological effects													
Verbal insults: No	24.5	29.8	27.9	2.14	.144	69.2	57.1	74.5	64.3	72.3	85.1	21.13	.001*
Physical threats: No	92.7	91.5	91.9	0.32	.573	92.3	89.3	91.5	88.4	93.3	95.0	5.93	.313
Sexual intimacy: No	67.0	72.3	70.4	2.07	.150	61.5	67.9	63.8	60.8	73.1	83.0	21.90	.001*
Stress: No	10.3	8.1	8.9	0.92	.337	0.0	17.9	12.8	13.6	6.3	4.3	16.05	.007^†^
Decision to avoid others: No	39.1	33.9	35.7	1.72	.190	7.7	42.9	21.3	33.2	42.9	36.9	14.96	.011*
Discriminatory experiences													
Aware of being gossiped about: No	33.0	46.4	41.7	11.14	.001*	23.1	53.6	44.7	45.2	42.4	34.0	8.12	.150
Aware of verbal insults/threats: No	19.3	28.6	25.4	6.96	.008*	15.4	35.7	29.8	30.2	23.9	18.4	8.98	.110
Aware of physical threats: No	7.7	13.4	11.4	4.82	.028*	7.7	17.9	6.4	13.6	11.3	9.2	4.09	.536
Discrimination by PLHIV: No	1.3	5.1	3.8	6.03	.014*	7.7	7.1	6.4	3.5	2.9	3.5	2.83	.726
Forced to change residence: No	2.6	7.6	5.9	7.00	.008*	7.7	14.3	6.4	8.0	3.8	4.3	7.95	.159
Gave social support													
Supported PLHIV: No	21.5	31.2	27.8	7.13	.008*	76.9	42.9	44.7	30.7	22.3	19.9	34.35	.000*
Emotional support: No	64.8	46.0	52.6	21.58	.000*	76.9	67.9	66.0	49.7	41.6	41.1	21.61	.001*
Physical support: No	49.4	68.8	62.0	24.37	.000*	92.3	71.4	72.3	60.3	60.1	59.6	9.23	.100
HIV disclosure: supportive family	43.3	36.5	38.9	3.00	.083	0.0	17.9	48.9	36.7	41.2	42.6	17.21	.004^†^
Member of PLHIV support group: No	88.4	76.0	80.3	14.81	.000*	69.2	85.7	89.4	81.4	79.0	78.0	4.85	.435
Feeling of power to…													
Influence local HIV projects: No	28.8	38.8	35.3	6.69	.010*	46.2	21.4	38.3	33.2	38.2	34.0	4.61	.466
Influence national programmes: No	45.5	59.1	54.4	11.34	.001*	69.2	53.6	55.3	59.3	50.4	52.5	4.83	.437
Rights abused: No	53.2	62.1	59.0	4.97	.026*	92.3	82.1	85.1	81.4	85.3	85.8	2.42	.788

* Significant *p* < 0.05,

† Fischer’s exact test.

PLHIV = people living with HIV, IHSM = internalised HIV-related stigma mastery, TB = tuberculosis

**Table 3:** Multiple regression analysis for factors predicting internalised HIV-related stigma mastery among 666 people living with HIV, 2014

Variable	Unadjusted OR, CI 95%	*p* value	Adjusted OR, 95% CI	*p* value
Demographic				
Gender: male	2.71, (1.95–3.76)	.000***	3.12, (1.98–4.88)	.000***
Age: ≤ 40 years	0.39, (0.28–0.53)	.000***	0.47, (0.33–0.69)	.000***
Self-employed: Yes	1.52, (1.12–2.07)	.007**	1.33, (0.93–1.90)	.118
Number of children <14years/household: ≤ 3	0.71, (0.51–0.99)	.046*	0.72, (0.49–1.05)	.086
Reason for HIV test: suspected tuberculosis	1.67, (1.14–2.44)	.009**	1.32, (0.85, 2.03)	.218
Psychological effects				
Stress: Yes	4.05, (2.06–7.94)	.000***	2.70, (1.27–5.72)	.010*
Fear of verbal insults: Yes	0.24, (0.16–0.35)	.000***	0.33, (0.19–0.56)	.000***
Fear of physical threats: Yes	0.25, (0.12–0.50)	.000***	0.80, (0.31–2.06)	.649
Fear of sexual intimacy: Yes	0.45, (0.32–0.64)	.000***	0.59, (0.38–0.90)	.014*
Decision to avoid others: Yes	1.55, (1.13–2.14)	.007**	1.40, (0.97–2.01)	.069
Social support to PLHIV				
Giving support: Yes	1.64, (1.16–2.33)	.005**	1.99, (1.04–3.79)	.038*
Emotional support: Yes	1.43, (1.05–1.95)	.022*	0.84, (0.52–1.36)	.476
Physical support: Yes	1.36, (0.99–1.86)	.057	0.90, (0.58–1.38)	.618
HIV disclosure: supportive family	1.39, (1.04–1.90)	.041**	1.70, (1.16–2.47)	.006**

Regression analysis variables were coded as follows: gender (0 = male; 1 = female); age (0 = ≤ 40 years; 1 = > 40 years); number of children < 14 years/household (0 = ≤3; >3 = 1); at HIV disclosure (0 = supportive family; 1= non-supportive); and the rest (0 = no; 1 = yes)

*OR* odds ratio, *CI* confidence interval, *PLHIV* people living with HIV

*** *p* < .0001,

** *p* < .001,

* *p* < .05

**Table 4:** Adjusted prevalence ratios for internalised HIV-related stigma mastery stratified by gender and age (40 years)

Variable	Men under 40 years (*n*= 86) PR 95% CI, *p* value	Men over 40 years (*n*=147) PR 95% CI, *p* value	Women, young (*n* = 201) PR 95% CI, *p* value	Women, old (*n* = 232) PR 95% CI, *p* value
Socio-demographic				
Self-employment	2.27 (1.73–3.60) .001**	1.07 (0.68–1.68) .784	0.92 (0.80–1.08) .330	1.18 (0.93–1.51) .171
≤ 3 children <14 years/household	0.63 (0.40–0.98) .040*	0.88 (0.54–1.42) .606	0.86 (0.70–1.04) .123	0.92 (0.70–1.20) .524
HIV test for suspected tuberculosis	1.34 (0.73–2.46) .348	0.82 (0.50–1.32) .401	1.32 (0.97–1.78) .074	1.14 (0.81–1.59) .456
Psychological variables				
Stress	1.23 (0.71–2.14) .445	1.95 (1.26–3.02) .003**	1.11 (0.94–1.30) .542	1.17 (0.72–1.89) .531
Verbal insult	0.40 (0.25–0.64) .000***	0.55 (0.34–0.91) .019*	0.85 (0.73–0.99) .045*	0.61 (0.47–0.79) .000***
Physical threat	1.30 (0.79–2.16) .302	1.36 (0.77–2.38) .281	0.90 (0.76–1.08) .252	0.89 (0.64–1.24) .504
Sexual intimacy	0.48 (0.31–0.73) .001**	0.68 (0.44–1.04) .072	1.11 (0.94–1.32) .192	0.82 (0.63–1.07) .144
Decision to avoid others	1.29 (0.83–2.00) .264	1.24 (0.75–2.06) .407	1.19 (1.00–1.43) .051	1.02 (0.77–1.37) .871
Social support to other people with HIV				
Giving support	2.13 (0.85–5.38) .109	0.63 (0.26–1.53) .304	1.02 (0.80–1.30) .872	1.50 (1.02–2.21) .039*
Emotional support	0.68 (0.32–1.45) .318	1.15 (0.60–2.23) .673	0.90 (0.73–1.12) .355	1.09 (0.78–1.52) .617
Physical support	0.89 (0.52–1.52) .671	1.12 (0.64–1.99) .686	0.97 (0.82–1.16) .764	0.85 (0.62–1.17) .310
HIV disclosure family support	1.32 (0.86–2.03) .196	2.24 (1.32–3.78) .003**	0.93 (0.80–1.08) .359	1.21 (0.95–1.55) .121

Adjusted for gender, age group, stress, fear of verbal insults, fear of sexual intimacy, giving support, and HIV disclosure family support

*** *p* < 0.001,

** *p* < 0.01,

* *p* < 0.05.

*PR* = prevalence ratio

**Table 5:** Correlation matrix for eight covariates for internalised HIV-related stigma mastery, 2014

	IHSM	SoE	SoS	FoFR	SoI	Gender	Age40	GIVES	DISCFAM
IHSM	1								
SoE	−.163**	1							
SoS	−.170**	.283**	1						
FoFR	−.181**	.220**	.258**	1					
SoI	−.260**	.289**	.386**	.557**	1				
Gender	.134**	−0.068	0.036	−.128**	−0.031	1			
Age40	−.195**	0.003	.132**	.091*	.158**	−.092*	1		
GIVES	.094*	−0.014	0.074	0.051	0.014	.158**	−.166**	1	
DISCFAM	.135**	0.050	−0.009	0.070	0.004	−.121**	−.356**	0.046	1

** *p* < 0.01 (two-tailed),

* *p* < 0.05.

Age40 = Age-related processes with cut-off at 40 years, DISCFAM = HIV disclosure family support, GIVES = giving emotional support to other PLWH, FoFR = fear of future rejection, IHSM = internalised HIV-related stigma mastery, SoE = social exclusion, SoI = social intimacy, SoS = social stress

**Table 6:** Factors, factor loadings, percentage of variance, and reliability statistics for principal component analysis with varimax rotation on internalised HIV-related stigma mastery items, 2014

Construct	Indicators	Factor loading (λ)	Error variance ε = (1-λ^2^)	Cronbach’s alpha (α)	Average variance extracted (AVE)= Σλ^2^/N	Construct reliability (CR) = Σλ^2^/(Σλ^2^+Σε)	Square root AVE
Internalised HIV-related stigma mastery (IHSM) 4 items	Q400_2 I feel no guilt	0.801	0.358	0.70	0.511	0.806	0.715
	Q400_8 Having HIV is not a sin	0.698	0.513				
	Q400_1 I feel no shame	0.685	0.531				
	Q400_3 I have no self-blame	0.667	0.555				
Social exclusion (SoE) 2 items	Q201 I was excluded at social gatherings	0.841	0.293	0.60	0.707	0.892	0.841
	Q202 I was excluded during family activities	0.841	0.293				
Social stress (SoS) 3 Items	Q216 I was never subjected to psychological pressure	0.813	0.338	0.60	0.547	0.726	0.740
	Q221_3 People think living with HIV is shameful	0.750	0.438				
	Q221_1 I experience discrimination for looking sick with symptoms	0.647	0.581				
Fear of future rejection (FoFR) 4 items PHYSICAGGRES	Q420_3 I was fearful of being physically assaulted	0.881	0.224	0.60	0.734	0.917	0.857
	Q420_2 I was fearful of being physically threatened	0.865	0.251				
	Q210_4 Who gossiped my neighbours	0.868	0.246				
NEIGHAGGRES	Q212_4 My Neighbours physically threatened me	0.812	0.341				
Fear of social intimacy (SoI) 5 items VERBINS	Q206 I was never aware of being verbally insulted, harassed or threatened	0.903	0.185	0.65	0.631	0.894	0.794
	Q211_4 Neighbours verbally insulted, harassed or threatened me	0.830	0.312				
Fear of sexual intimacy	Q217 I have no experiences of sexual rejection	0.778	0.395				
	Q423 Afraid of sexual intimacy for living with HIV	0.751	0.436				
	Q420_1 Fear of verbal insults/harassment/threats	0.693	0.519				
Give support to other PLWH (GIVES) 2 items	Q620_1 I provided emotional support such as sharing personal stories to other PLHIV	0.909	0.174	0.74	0.826	0.905	0.909
	Q619 Provide support to other PLHIV	0.909	0.174				
HIV disclosure family support (DISCFAM) 2 items	Q802 I or someone else with my consent told children in my family that I was living with HIV	0.929	0.137	0.84	0.863	0.927	0.929
	Q823 Children in my family were supportive when they first knew that I was living with HIV	0.929	0.137				

PLHIV = people living with HIV. Kaiser-Meyer-Olkin (.50 to .70) and Bartlett’s test of sphericity .124 to .574 (p ≥.001). Commonality for items = λ^2^

**Table 7:** Structural equation model constructs, directions of relationships, and estimated unstandardized and standardized regression coefficients for internalized HIV-related stigma, 2014

Construct	Path	Construct	Unstandardized regression coefficient estimates	Standardized regression coefficient estimates	SE	CR	*P*	T value	Result
SoS	<---	SoE	0.325	0.284	0.042	7.66	***	6.76	Supported
SoS	<---	GIVES	0.035	0.078	0.016	2.10	.036	4.88	Supported
Age40yrs	<---	SoS	0.372	0.144	0.091	4.08	***	1.58	Supported
FoFR	<---	Gender	−0.053	−0.126	0.015	−3.44	***	−8.40	Supported
Age40yrs	<---	Gender	−0.123	−0.119	0.037	−3.30	***	−3.22	Supported
Age40yrs	<---	DISCFAM	−0.387	−0.364	0.038	−10.27	***	−9.58	Supported
FoFR	<---	SoE	0.180	0.149	0.046	3.90	***	3.24	Supported
FoFR	<---	SoS	0.232	0.221	0.04	5.78	***	5.53	Supported
Age40yrs	<---	GIVES	−0.162	−0.142	0.041	−3.96	***	−3.46	Supported
SoI	<---	FoFR	0.562	0.465	0.038	14.68	***	12.24	Supported
SoI	<---	SoS	0.279	0.219	0.041	6.74	***	5.34	Supported
SoI	<---	SoE	0.180	0.124	0.046	3.89	***	2.70	Supported
SoI	<---	Age40	0.042	0.086	0.015	2.83	.005	5.73	Supported
IHSM	<---	FoFR	−0.052	−0.038	0.061	−0.86	.392 ns	−0.62	Not supported
IHSM	<---	GIVES	0.041	0.063	0.024	1.71	.088 ns	2.63	Not supported
IHSM	<---	Age40	−0.051	−0.090	0.023	−2.25	.024	−3.91	Supported
IHSM	<---	SoS	−0.096	−0.066	0.059	−1.63	.104 ns	−1.12	Not supported
IHSM	<---	SoI	−0.200	−0.173	0.053	−3.75	***	−3.26	Supported
IHSM	<---	Gender	0.068	0.117	0.022	3.12	.002	5.32	Supported
IHSM	<---	DISCFAM	0.073	0.121	0.024	3.09	.002	5.04	Supported
IHSM	<---	SoE	−0.138	−0.083	0.064	−2.15	.032	−1.30	Supported

Age40 = age-related processes with cut-off at ≤ 40 years, HIV disclosure family support, GIVES = giving emotional support to others, FoFR = fear of future aggression, IHSM = internalized HIV-related stigma mastery, SoE = social exclusion, SoI = social intimacy, SoS = social stress

*** *p* < 0.001;

** *p* < .01

* *p* < 0.05;

ns = not significant (*p* > 0.05.)

**Table 8:** Total, direct, and indirect effects with their 95% bias-corrected bootstrap confidence intervals, 5000 replicates for internalized HIV-related stigma mastery, 2014

Hypothesis	Total effects (c)	Direct effects (c′)	Indirect effects (ab)	Lower 2.5%	Upper 97.5%	Mediation
SoE -> SoS -> AGEGP	.041***		.041***	.060	.200	Full
SoE -> SoS -> AGEGP -> FoFR	.212***	.151**	.061***	.030	.140	Partial
SoE -> SoS -> AGEGP -> FoFR-> SoI	.289***	.124**	.165***	.140	.340	Partial
SoE -> SoS -> AGEGP -> FoFR-> SoI -> IHSM	−.163***	−.083 ns	−.079***	−.210	−.080	Full
DISCFAM -> AGEGP -> SoI	−.031**		−.031**	−.030	−.004	Full
DISCFAM -> AGEGP -> IHSM	.159***	.121**	.039*	.003	.045	Partial
GENDER -> FoFR-> SoI	−.073***		−.073***	−.057	−.020	Full
GENDER -> AGEGP -> IHSM	.145***	.117***	.028***	.008	.029	Partial
GIVES -> SoS -> AGEGP	−.130***	−.142***	.011*	.002	.025	Partial
GIVES -> SoS -> AGEGP -> FoFR	.017*		.017*	.003	.040	Full
GIVES -> SoS -> AGEGP ->FoFR-> SoI	.041 ns		.041 ns	−.001	.046	No
GIVES -> SoS -> AGEGP ->FoFR-> SoI -> IHSM	.060***	.063 ns	−.003 ns	−.017	.012	No
SoS -> AGEGP->FoFR-> SoI	.333**	.220**	.113***	.079	.218	Partial
SoS -> AGEGP->FoFR-> SoI ->IHSM	−.145***	−.066 ns	−.079***	−.175	−.069	Full
AGEGP ->FoFR-> SoI -> IHSM	−.105*	−.090*	−.015**	−.019	−.002	Partial
FoFR-> SoI -> IHSM	−.118**	−.038 ns	−.081***	−.189	−.049	Full

Indirect effects were claimed if lower and upper bounds of calculated 95% bias-corrected bootstrap confidence intervals from 5000 bootstrap replicates did not include zero. A decision of partial mediation was made when all three of total, direct, and indirect were significant, and full mediation when direct effects were non-significant. The direct effects (c′) of giving support on age40 = −.142*** had an opposite sign to indirect effects (ab) via social stress = .011*; these are behaviours of a suppressor variable

*** *p* < 0.001,

** *p* < .01,

* *p* < 0.05;

ns = not significant (when *p* > 0.05). AGEGP = age 40 years-related processes, DISCFAM = HIV disclosure family support, FoFR = fear of future rejection, GIVES = gives emotional support to peers, IHSM = internalized HIV-related stigma mastery, SoE = social exclusion, SoS = social stress

## Data Availability

The data set used for analysis is attached but it is confidential.

## Abbreviations

IHSM: Internalized HIV-related stigma mastery
PLHIV: People living with HIV
PLHIV Stigma Index: The People Living with HIV Stigma Index questionnaire version 2008
